# Influence of Different Nicotine Sources on Exercise-Driven Immune Responses of Peripheral Blood Monocytes

**DOI:** 10.3390/toxics13060472

**Published:** 2025-06-02

**Authors:** Paul N. Axt, Theresa Mohr, Armin Steffen, Kirstin Plötze-Martin, Nele Jagodzinski, Sabine Bohnet, Daniel Drömann, Karl-Ludwig Bruchhage, Klaas F. Franzen, Ralph Pries

**Affiliations:** 1 Medical Clinic III, University Hospital Schleswig-Holstein, 23538 Lübeck, Germanydaniel.droemann@uksh.de (D.D.);; 2 Department of Otorhinolaryngology, University Hospital Schleswig-Holstein, 23538 Lübeck, Germany

**Keywords:** smoking, exercise, monocytes, PD-L1, growth hormone

## Abstract

Tobacco smoking is closely associated with pro-inflammatory immunological alterations, whereas regular physical exercise is well known to lower systemic inflammations and related immune cell activities. The combined effects of smoking, nicotine pouch use, vaping, and exercise on individual immunological responses remain incompletely understood, especially in view of alternative nicotine delivery systems. In this study, we analyzed the immediate impact of different nicotine sources on exercise monocyte subsets in 16 human subjects using a four-arm cross-over design. Distribution of circulating CD14/CD16 monocyte subsets and expression of the monocytic checkpoint molecule PD-L1 (programmed cell death ligand-1) were analysed via whole blood flow cytometry measurements. Plasma cytokines were evaluated using membrane-based cytokine arrays and enzyme-linked immunosorbent assays (ELISA). Data revealed significant distributions of circulating monocytes subsets in response to nicotine consumption and physical stress. In contrast, exercise-driven increased monocytic PD-L1 was clearly attenuated following the consumption various nicotine delivery systems. Furthermore, significantly increased plasma growth hormone levels were detected in response to physical stress in combination with cigarette consumption. Our data clearly illustrates a significant influence of nicotine consumption on the cellular characteristics of circulating monocyte subsets and on proper exercise-driven immune responses within a short period of time, which makes the widespread trivialization of alternative nicotine sources questionable.

## 1. Introduction

It is well established that tobacco smoking is closely associated with immunological alterations and elevated abundances of pro-inflammatory cytokines, such as tumor necrosis factor a (TNF-a) and interleukins (IL)-1, 6 and 8 [1]. Long-term smoking can lead to reduced activities of T-lymphocytes and diminished levels of immunoglobulins and natural killer (NK) cells [2]. Even a single bout of tobacco smoking can trigger circulating leukocytes to amplify inflammatory responses [3]. Furthermore, tobacco consumption via smoking, heating, and vaping is closely associated with the development of different diseases such as atherosclerosis, cardiovascular disease, and different types of cancer [4]. In recent years, new nicotine delivery systems and nicotine products have been developed, most of them being promoted as less harmful alternatives to regular tobacco smoking. However, it is becoming increasingly clear, that these products are not considered to be low risk, leading to adverse effects such as endothelial dysfunction, oxidative stress, and systemic inflammation and are even associated with an increased risk of lung cancer [5,6,7]. In this context, it has recently been shown that anti-oxidative and pro-inflammatory biomarkers of human monocytes were induced by cigarette smoke as well as by alternative next-generation nicotine products [8].

However, knowledge of the impact of tobacco smoke and different nicotine delivery systems (e.g., e-cigarettes, etc.) on the immunological cellular characteristics of human monocytes remains limited. Similarly, data on the effect on systemic inflammation after using a pouch are scarce, especially in direct comparison to combustible cigarettes, e-cigarettes, and sham vaping or smoking. In contrast, regular exercise can lower systemic inflammation associated with an increased release of myokines from skeletal muscle and a reduction of membrane-bound immune receptors involved in immune cell activity and release of inflammatory cytokines [9]. Moreover, it has recently been shown that interval exercise increases the abundance of circulating PD-1+ CD8 central memory T cells as well as soluble PD-L1 [9]. Immune checkpoint molecules PD-1 (programmed death-1) and PD-L1 (programmed death ligand-1) play a critical role in maintaining physiological health by downregulating inflammatory responses and maintaining immune system balance [10]. The independent interplay and contribution of all these processes on the individual immunologic situation is still not fully understood, especially in view of alternative nicotine delivery systems used for nicotine consumption, followed by physical exercise and a final resting phase on immunological responses regarding peripheral blood monocyte subsets. Therefore, we analyzed the distribution of circulating CD14/CD16 monocyte subsets as well as the expression of checkpoint molecule PD-L1 (programmed cell death ligand-1) using flow cytometry. Furthermore, expression patterns of a variety of different plasma cytokines were evaluated. The aim of this study was to increase our understanding of the temporal immunological responses to smoking, vaping, and physiological exercise, and their mutual interactions at the cellular level. Therefore, we hypothesize that different forms of acute nicotine consumption modulate the exercise-induced immune response of peripheral blood monocytes, particularly with respect to PD-L1 expression and monocyte subset distribution.

## 2. Materials and Methods

### 2.1. Ethics Statement

All individuals in our study were examined at the Department of Medical Clinic III, University Hospital Schleswig-Holstein, Campus Luebeck, and provided written informed consent. The study was approved by the local ethics committee of the University of Luebeck (approval number 2022-485, 13 October 2022) and is registered in DRKS (DRKS00030564). The study was conducted in accordance with the ethical principles for medical research formulated in the WMA Declaration of Helsinki.

### 2.2. Study Cohort and Design

A total of 16 healthy, normal weight test subjects were included. Participants were screened for the following diseases: hypertonus, diabetes mellitus type, thrombosis, pulmonary embolisms, thyroid dysfunctions, coagulopathy and congenital heart disease. One participant suffered from familial hypercholesterolemia. Another participant reported to have developed a diabetes mellitus type I shortly after the end of measurements. None of the other participants reported any diseases. One participant took rosuvastatin and flunarizine. Another participant took minocycline, and two female participants used a birth control pill. Participants drank on average 2.25 (±2.65) bottles of beer per week. Nobody reported to having ever used performance enhancing drugs. The average weekly amount of exercise, smoking behavior, and basic values of lung function were assessed. Sex specific values are presented in Table 1.

In this four-arm crossover study, 16 healthy occasional smokers were recruited among students of Lübeck University. Exclusion criteria encompassed non-smokers, individuals unable to stay smoke-free for 48 h or those with a Fragström test score of 3 or higher. The Fragström test is a self-administered questionnaire aimed at evaluating an individual’s dependence on smoking in everyday life. Additionally, subjects with a history of cardiovascular or pulmonary diseases, a reduced Tiffeneau index during initial spirometry, those under 18 years of age, individuals with obesity, an ongoing pregnancy, severe mental health issues, and anyone with abnormal results from physical examinations were excluded. The Tiffeneau index was measured during the initial spirometry to assess airway obstruction. This index serves as a pulmonary function parameter that quantifies the ratio of a subject’s forced expiratory volume in one second (FEV1) to their forced vital capacity (FVC). A ratio below 0.7 generally indicates the presence of obstructive lung conditions such as asthma or chronic obstructive pulmonary disease (COPD). Throughout the four sessions, subjects were exposed to one of four interventions (A: control (vaping an e-cigarette without liquid); B: smoking a combustible cigarette; C: vaping an e-cigarette with a tobacco flavor; D: using a nicotine pouch). They also underwent spiroergometric testing, with blood samples collected at various points during the study.

**Figure 1:**
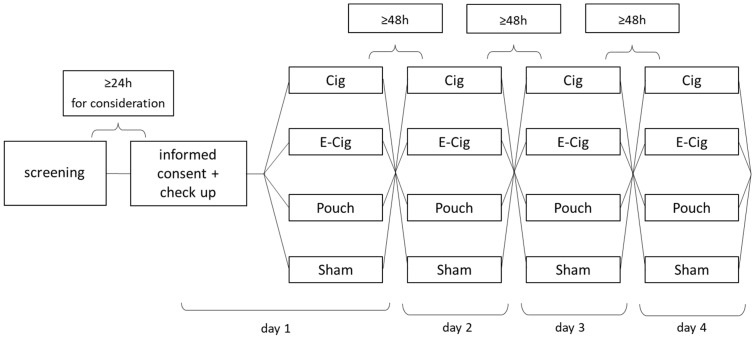
Flowchart and study design for each participant. Every device is tested once, whereas the order is randomly determined at the beginning of each measurement day (Cig—smoking a combustible cigarette; E-Cig—vaping an e-cigarette with tobacco flavor; Pouch—consumption of a nicotine pouch; Sham—vaping an e-cigarette without liquid as a control).

Figure 1 displays a flowchart of the study. To be taken into analysis, each test subject needed to participate in all four study arms. The order of the four different study arms was randomized by drawing a lot each measurement day. To avoid distortion of measurements, test subjects were instructed to remain nicotine free 48 h- and alcohol free 24 h prior to each measurement. On measurement days they were asked not to consume caffeine and to stick to a light breakfast. Measurements were scheduled at least 48 h apart to provide the required nicotine washout period. In addition, measurements always took place in the mornings to reduce possible bias due to circadian rhythm.

### 2.3. Consumption of Nicotine Delivery Systems and Combustible Cigarette

Analyzed nicotine delivery products were: (a) e-cigarettes with tobacco flavor (Device: DIPSE-eGO-cigarette, Liquid: LiQueen Italian tobacco nicotine: 24 mg/mL), (b) combustible cigarettes (Marlboro Gold nicotine: 0.5 mg/cigarette), (c) nicotine pouches (NICO^TM^ Whip Alpine mint, nicotine: 10 mg/g, 1 pouch = 800 mg) and (d) e-cigarettes without liquid (Device: DIPSE-eGo-cigarette; Liquid: none nicotine: 0 mg/mL) as control (sham). The different types of nicotine consumption and nicotine concentrations of the various products lead to considerable differences in nicotine concentration (in ng/mL) and kinetics in the blood. Based on parallel work [11,12], we know that cigarettes lead to the highest nicotine concentration in the blood. The e-cigarette has comparable kinetics with an overall flatter concentration curve with the peak around 6 min after consumption. The maximum nicotine concentration of the pouch is between that of the e-cigarette and the cigarette but only reaches the maximum concentration after about 20 min and thus with a significant delay compared to inhaled nicotine consumption. Consumption was carried out following a protocol to grant similar intra- and inter-individual substance uptakes. Combustible cigarettes: one cigarette was to be inhaled completely in 5 min. E-cigarette and sham smoking: Vaping of a total of 10 puffs in 5 min with one inhale for 5 s every 30 s. Nicotine pouch: Inserting a nicotine pouch between palate and upper lip for 5 min. All devices were consumed outside.

### 2.4. Ergospirometry

In this study, subjects engaged in bicycle ergospirometry to assess various spirometric parameters using a Cosmed device (Omnia software version 1.6.10). The exercise protocol began with a two-minute warm-up at a load of 25 W, followed by a ramp protocol starting at the same intensity. The goal was to reach a predetermined target load after 11 min, calculated according to the Jones protocol. This maximum load was then used as a reference for the next three testing sessions. Each subject was positioned on the ergometer and wore a snug mask to measure oxygen and carbon dioxide output, as well as tidal volume and breathing frequency. Blood pressure was monitored through an automatic system, while heart rate and rhythm were continuously tracked using a 12-lead ECG. Monitoring continued for an additional five minutes during the recovery phase. Testing was halted if subjects experienced significant fatigue, severe shortness of breath, chest discomfort, or dizziness (subjective criteria) or if specific objective criteria were met, such as notable ST segment shifts exceeding 0.2 mV, signs of progressive blockages, arrhythmia, substantial declines in heart rate or blood pressure or if systolic pressure rose above 260 mmHg.

### 2.5. Staining of Monocyte Subsets in Whole Blood

Blood was drawn by venipuncture into a sodium citrate containing S-Monovette (Sarstedt; Nümbrecht, Germany). Within 4 h after blood collection, 20 µL of citrate blood was diluted in 80 µL PBS. Blood cells were then stained with following antibodies: CD45-PE, CD14-FITC, CD16-BV-510, HLA-DR-APC-Cy7, and PD-L1-APC, (all from Biolegend, San Diego, CA, USA). After staining in the dark for 25 min, 650 µL RBC Lysis Buffer (Biolegend) were added to the samples and incubated for another 20 min. Subsequently, the suspension was centrifuged at 400× *g* for 5 min and the supernatant was discarded. Cell pellet was resuspended in 100 µL fresh PBS and used for FACS analysis.

### 2.6. FACS Analysis

Using a MACSQuant 10 flow cytometer (Miltenyi Biotec, Bergisch-Gladbach, Germany) the flow cytometric measurements were performed, and the generated data was analyzed using the FlowJo software version 10.0 (FlowJo, LLC, Ashland, OR, USA). All antibody titrations and compensations were performed beforehand. For whole blood measurements, at least 100,000 CD45^+^ leukocytes were analyzed. Gating of monocyte subsets was performed as described before [13]. In short, CD45 was used as a pan leukocyte marker to facilitate whole blood measurement and monocytes were first roughly gated by their FSC/SSC characteristics and the positivity for CD14 and CD16. Neutrophil granulocytes, NK-cells and B-cells were excluded by means of HLA-DR which is specific for monocytes. Remaining monocytes were then then categorized in CD14^++^CD16^−^ (classical), CD14^++^CD16^+^ (intermediate) and CD14^dim+^CD16^+^ (non-classical) monocytes.

### 2.7. Cytokine Analysis

The collected plasma samples were instantly frozen with liquid nitrogen and preserved at −80 °C. Analyses of plasma cytokines and chemokines were performed using membrane-based Proteome Profiler^TM^ Human XL cytokine arrays (R&D Systems, Minneapolis, MN, USA) as recommended by the supplier. Expression was visualized using an enhanced chemiluminescence detection kit (R&D Systems, Minneapolis, MN, USA). An initial cytokine screening using membrane-based arrays was performed on plasma samples from one representative subject to identify potentially regulated targets. Based on these results, plasma growth hormone (GH) levels were subsequently quantified by ELISA across the entire study cohort (n = 16). Semiquantitative analysis was performed by measuring the density of the bands using an iBright CL 1000 biomolecular imager (Invitrogen, Carlsbad, CA, USA). Plasma concentrations of growth hormone (GH) were assessed from citrate-plasma samples and were determined by enzyme-linked immunosorbent assays (ELISA) according to manufacturer’s protocols (R&D Systems, Minneapolis, MN, USA).

### 2.8. Statistical Analyses

Statistical analyses were performed using Graph Pad Prism (Graph Pad Prism 6.01 for Windows, Graph Pad Software, San Diego, CA, USA). The mean and standard error (SEM) are presented. The differences between groups were determined after testing for Gaussian distribution (normality tests), and applying paired student’s *t*-test of different pairs of measured conditions of our study group. The correlation between parameters was calculated using multivariate regression with the Pearson correlation coefficient. *p* < 0.05 (*), *p* < 0.01 (**), *p* < 0.001 (***). Additional statistical details are given in the respective figure legends, where appropriate.

## 3. Results

### 3.1. Monocyte Subset Distribution and PD-L1 Expression

**Figure 2:**
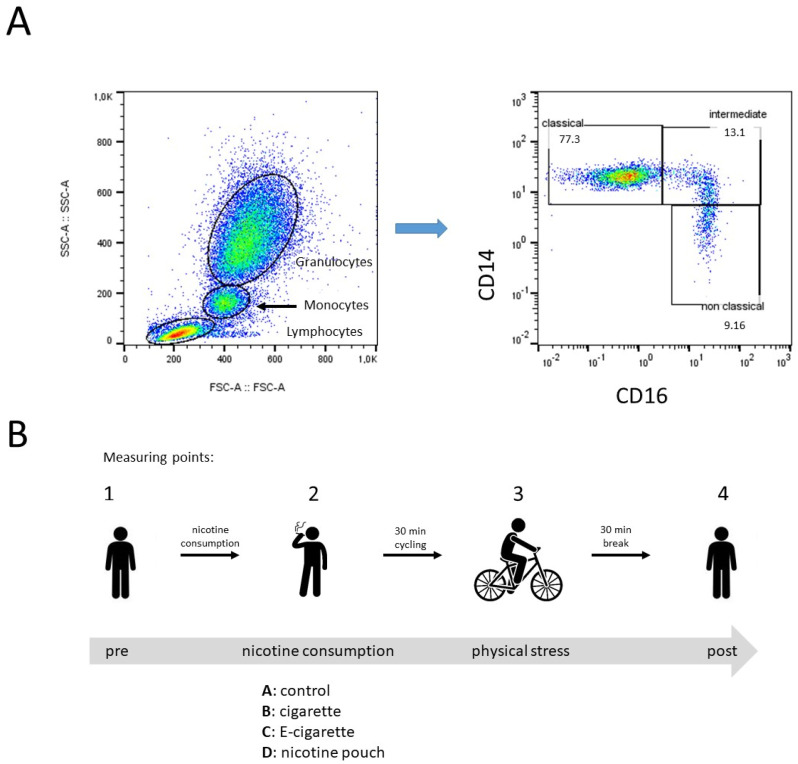
Study scheme and flow cytometric analysis of peripheral blood monocyte subsets. (**A**) Representative example gating scheme of monocyte subset analysis with regard to the forward scatter (FSC)/sideward scatter (SSC) characteristics and the CD14/CD16 expression levels of circulating classical (CM), intermediate (IM) and non-classical monocytes (NCM). (**B**) Study scheme illustrates the measuring points (1) before nicotine consumption, (2) after nicotine consumption, (3) after physical stress, and (4) after 30 min break. Measuring point 2 can be distinguished in (A) control, (B) cigarette, (C) e-cigarette, and (D) nicotine pouch.

Whole blood flow cytometric measurements were performed to investigate the individual abundances of peripheral blood CD14/CD16 monocyte subsets and associated expression levels of checkpoint molecule PD-L1 during the course of our study. Gating of monocyte subsets was conducted as described previously [13] (Figure 2A).

Figure 2B illustrates the course of our study and the corresponding whole blood measuring points: (1) before nicotine consumption, (2) after nicotine consumption, (3) after physical stress, and (4) after 30 min break, whereas measuring point 2 can be distinguished in (A) control, (B) cigarette, (C) e-cigarette, and (D) nicotine pouch.

**Figure 3:**
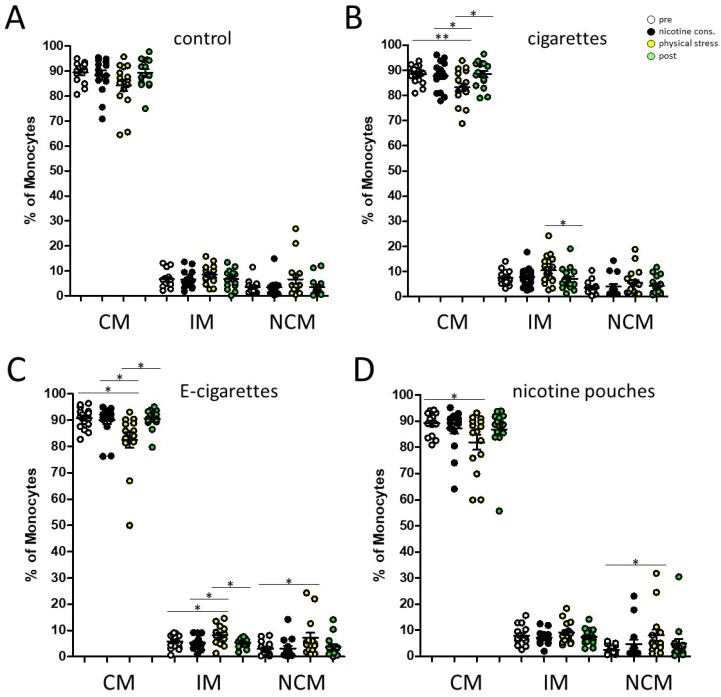
Flow cytometric analysis of peripheral blood monocyte subsets. Percentages of circulating classical (CM), intermediate (IM) and non-classical monocytes (NCM) in the peripheral blood of our cohort during the course of our study (pre; nicotine consumption; physical stress; post). The different parameters (**A**) control (consumption of nicotine free e-cigarette), (**B**) cigarette, (**C**) e-cigarette, and (**D**) pouch where investigated with regard to the measuring point ‘nicotine consumption’. *: *p* < 0.05; **: *p* < 0.01.

**Figure 4:**
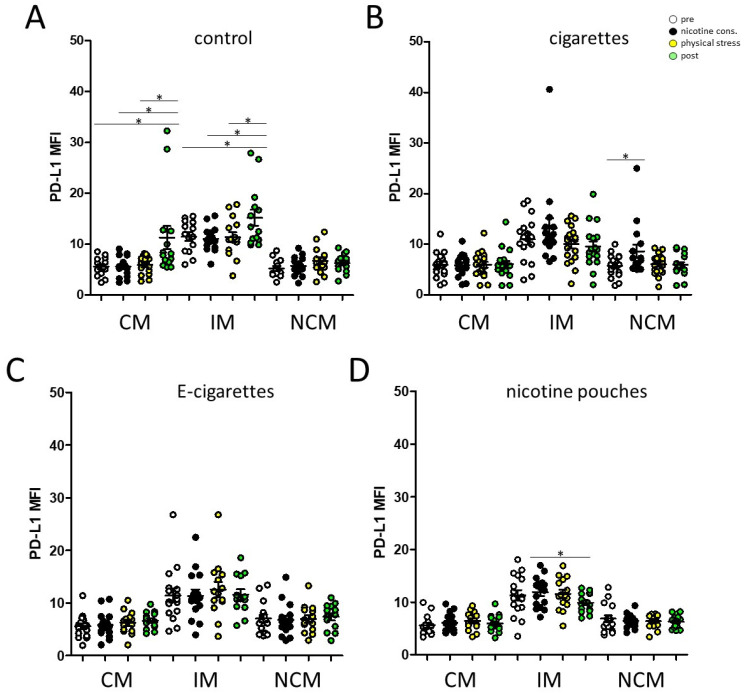
Checkpoint molecule PD-L1 on peripheral blood monocyte subsets (CM: classical monocytes; IM: intermediate monocytes; NCM: non-classical monocytes) during the course of our study (pre; nicotine consumption; physical stress; post). Different parameters (**A**) control (consumption of nicotine free e-cigarette), (**B**) cigarette, (**C**) e-cigarette, and (**D**) pouch were investigated with regard to the measuring point ‘nicotine consumption’, respectively. *: *p* < 0.05. MFI: mean fluorescence intensity.

Data revealed significantly decreased percentages of circulating classical (CM) monocytes partially accompanied with significantly increased abundances of intermediate (IM) and non-classical (NCM) monocytes in response to nicotine consumption and physical stress at measuring point 3 (Figure 3). Next, flow cytometric analyses of PD-L1 expression levels on circulating monocyte subsets were performed (Figure 4).

Data revealed significantly increased PD-L1 expression levels on circulating classical and intermediate monocytes in response to physical stress at measuring point 4 in our control group, which could not be observed after a previous consumption of the different nicotine sources cigarette, e-cigarette, and pouch (Figure 4).

### 3.2. Cytokine Secretion upon Smoking and Acute Exercise

Next, screening for potential factors which might be involved in the observed redistributions and immune responses of circulating CD14/CD16 monocyte subsets was carried out.

**Figure 5:**
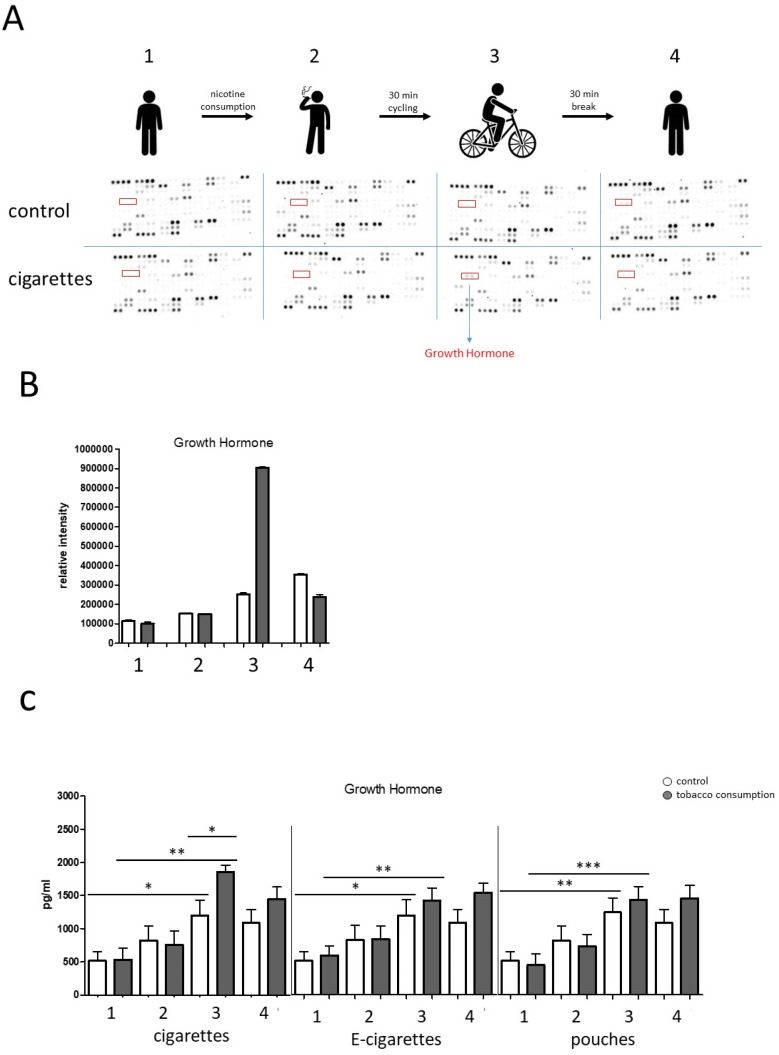
Cytokine screening during the course of our study. (**A**) Raw images of membrane-based cytokine arrays of plasma samples of one tested individual revealed increased growth hormone levels in response to cigarette smoking and physical stress at measuring point 3 compared to the non-smoking control. (**B**) Semiquantitative analysis was conducted by measuring the density of the growth hormone dots. (**C**) ELISA measurements of growth hormone (GH, pg/mL) plasma concentrations during the course of our study. Data revealed significantly increased GH levels in response to physical stress in combination with the consumption of the different nicotine sources such as cigarette, e-cigarette, and pouch (measuring point 3) compared to the control (consumption of nicotine free e-cigarette). *: *p* < 0.05; **: *p* < 0.01; ***: *p* < 0.001.

Therefore, plasma levels of 105 different cytokines and chemokines were measured in samples from one individual during the course of our study using a membrane-based human cytokine antibody array. Semiquantitative analyses of the density of the dots revealed relatively homogeneous cytokine expression patterns with regard to the distinct measurement points of our study (Figure 5A).

However, semiquantitative analysis by measuring the density of the dots revealed increased expression levels of growth hormone (GH) particularly in response to cigarette smoking and physical stress at measuring point 3 compared to the non-smoking control. (Figure 5A,B). In order to quantify and evaluate the individual plasma GH levels during the course of our study, ELISA measurements of the entire cohort under all tested conditions were performed. Data revealed significantly increased GH levels in response to physical stress under all tested conditions compared to the starting situation before nicotine consumption (pre) (Figure 5C). Furthermore, we observed significantly increased plasma GH levels in response to physical stress in combination with cigarette consumption compared to the non-smoking/physical stress control (Figure 5C).

## 4. Discussion

In addition to inducing genomic alterations [14], tobacco consumption is known to affect the abundance and functionality of different immune cell populations, such as CD8^+^ T lymphocytes [15].

We have previously shown that THP-1 monocytes stimulated with aqueous extracts from various nicotine delivery systems, such as e-cigarettes, nicotine pouches, and heat-not-burn products (e.g., IQOS), revealed distinct alterations of adhesion molecules and cytokine secretion patterns [16]. Therefore, the present study aimed to elucidate the impact of different nicotine sources (like combustible cigarette, e-cigarette and pouches) on exercise-driven effects in primary peripheral blood monocyte subsets to better understand the in vivo situation of these two contradictory ‘activities’. Our data revealed significantly reduced levels of classical monocytes, accompanied by increased percentages of CD16^+^ monocytes, following nicotine consumption. These alterations were not observed in the control arm without nicotine consumption. The extent of the changes in monocyte concentrations caused by the different nicotine products was in line with the expected nicotine concentration with the greatest change for the cigarette smoking, followed by pouch use, and then e-cigarette use. Similarly, it has been shown in mice that exposure to cigarette smoke resulted in an altered composition of pulmonary macrophages with elevated CD11b^+^ subpopulations including monocyte-derived alveolar macrophages [17]. However, considering the small sample size of our cohort, data must be interpreted with caution. There is also evidence on an exercise alone driven effect on the distribution of circulating monocyte subsets, whereas this study was also based on a small cohort of only 15 male individuals [18].

With respect to the expression levels of checkpoint molecule PD-L1 on primary monocyte subsets, we observed likewise inhibitory effects on the cellular response of the analyzed nicotine sources. Checkpoint molecule PD-L1 is an important regulator and limiter of different aspects of immune responses and present on different types of immune cells including B cells, T cells, dendritic cells, and monocytes [19,20,21,22].

Data revealed significantly increased PD-L1 expression levels on classical and intermediate monocytes in the non-smoking control during the course of our study, most likely due to the physical stress and the related oxygen deficiency. Intermitted hypoxia has been associated with an upregulation of HIF-1α and increased expression levels of checkpoint molecule PD-L1 on monocytes and T cells [23]. Similarly, systemic hypoxia in patients with obstructive sleep apnoea syndrome (OSAS) is well known to trigger alterations in different immune cells such as lymphocytes, NK cells and monocytes [13,24,25,26,27,28]. Increased PD-L1 expression levels on circulating monocytes in response to physical exercise were strongly attenuated following consumption cigarettes, e-cigarettes, and nicotine pouches, respectively.

These data strongly underline the proposed role of nicotine as an inhibitor of proper immune responses and cytokine secretion [29]. In this context, nicotine is suggested to act as a cholinergic substance by stimulating the release of acetylcholine, either directly by stimulating nicotinic receptors or indirectly by inhibiting cholinesterase [30]. The impact of nicotine consumption on distinct immune functions has been thoroughly investigated, whereas potential effects on plasma cytokine patterns remain underexplored. In this work, we evaluated the growth hormone (GH) plasma concentration profiles during the short-term course of our study. Data revealed significantly increased GH levels in response to physical stress under all tested conditions compared to the starting situation before nicotine consumption (pre). Additionally, even higher significant levels of GH were measured upon prior cigarette consumption, which corroborates earlier observations of increased circulating levels of growth hormone upon nicotine consumption and sporting activities. Human growth hormone is secreted in response to different stimuli, whereas sleep and exercise are particularly strong stimulators. GH is involved in various physiological processes in the human body, including the turnover of muscle, bone and collagen, and the regulation of fat metabolism [31,32]. Moreover, it has been shown that growth hormone acts as a human macrophage-activating factor, priming monocytes for enhanced superoxide production [33,34] and may alter physiologically relevant processes such as proteolysis and antigen presentation [35].

With respect to limitations, the present study focused on acute immune responses following short-term nicotine exposure and physical stress. We acknowledge that the transient nature of the observed immunological alterations limits conclusions regarding long-term biological significance. Nonetheless, the rapid shifts in monocyte subset distribution and PD-L1 expression provide valuable insights into the immediate modulation of innate immunity, which may be particularly relevant in the context of acute inflammatory challenges such as infections, physical exertion, or vaccination. Given the exploratory nature of this pilot study, no formal a priori power calculation was performed. The sample size was determined based on feasibility and the aim to generate initial insights into the acute immunological effects of different nicotine delivery systems. We acknowledge this as a methodological limitation and explicitly consider the present work as pilot study. Future studies with extended follow-up periods and larger, statistically powered cohorts will be necessary to validate and further explore the implications of these findings in chronic exposure scenarios. In additions, larger cohorts will be essential to assess their generalizability.

Taken together, our data indicates a significant influence of nicotine consumption on the cellular characteristics of circulating monocyte subsets within a short timeframe. Furthermore, proper exercise-driven immune responses are significantly altered following consumption of all analyzed nicotine sources, raising concerns about the widespread trivialization of alternative nicotine sources. Further investigations involving larger study cohorts will help to better understand the risk of different nicotine sources, delivery methods, and concentrations on the immunologic situation in the human body. We also recommend future investigations comparing these nicotine products in the absence of physical activity and including the newest generations of electronic nicotine delivery systems (ENDS).

## Figures and Tables

**Table 1:** Clinical characteristics of the analyzed study cohort.

Sex	All (n = 16)	Male (n = 10)	Female (n = 6)
Age [years]	24.0 ± 3.8	24.6 ± 4.3	23.0 ± 2.8
Weight [kg]	72.2 ± 9.3	75.5 ± 9.1	66.7 ± 7.4
Height [cm]	179.4 ± 8.8	184.0 ± 7.1	171.7 ± 4.9
BMI [kg/m^2^]	22.4 ± 1.9	22.3 ± 2.2	22.6 ± 1.4
Exercise per Week [h]			
<3/3–10/>10	2/9/1	2/6/2	0/5/1
Tiffeneau Index	0.79 ± 0.09	0.81 ± 0.09	0.76 ± 0.09
Cigarettes per week	4.0 ± 4.1	4.8 ± 4.2	2.9 ± 4.0
Fagerström Test for Nicotine Dependence [points]	0.0 ± 0.0	0.0 ± 0.0	0.0 ± 0.0

## Data Availability

Data available upon request.
